# Clinical, psychosocial and cognitive outcomes following VBAC: a protocol for a sub-national cohort study in Saudi Arabia (2019–2024)

**DOI:** 10.1136/bmjopen-2025-114754

**Published:** 2026-10-13

**Authors:** Asma A Aldawsari, Sufyan Alzomia, Nouf F Alanizi, Seena Thomas, Bienvenido Paras, Lubna A Alnasser, Abdullah Alghamdi, Hattan F Dagestani, Ann-Beth Moller, Laith Hussain-Alkhateeb

**Affiliations:** 1 Department of Population Health, King Abdullah International Medical Research Center (KAIMRC), Riyadh, Saudi Arabia; 2 King Saud Bin Abdulaziz University for Health Sciences (KSAU-HS), Riyadh, Saudi Arabia; 3 Ministry of National Guard Health Affairs (MNGHA), Riyadh, Saudi Arabia; 4 Women’s Health Hospital, Department of Nursing, National Guard Health Affairs, Riyadh, Saudi Arabia; 5 King Abdullah Specialized Children’s Hospital (KASCH), National Guard Health Affairs, Riyadh, Saudi Arabia; 6 Department of Pediatrics, King Abdullah Specialized Children Hospital, Riyadh, Saudi Arabia; 7 Department of OBGYN, National Guard Health Affairs, Riyadh, Saudi Arabia; 8 School of Public Health and Community Medicine, Sahlgrenska Academy, University of Gothenburg Institute of Medicine, Gothenburg, Sweden

**Keywords:** Pregnancy, Pregnant Women, GYNAECOLOGY

## Abstract

**Abstract:**

**Introduction:**

Over recent decades, global caesarean section (CS) rates have risen sharply. In Saudi Arabia, this trend has become particularly noticeable, with the national CS rate reaching 28.1% in 2024. Vaginal birth after CS (VBAC) offers a safe alternative to elective repeat CS (ERCS) for many women with previous CS. VBAC is linked to lower maternal complications, faster recovery and lower healthcare costs. However, despite its known advantages, there is a significant lack of strong evidence connecting VBAC practices to potential clinical, psychosocial and cognitive health outcomes for mothers and children in the Saudi context. This study protocol presents a detailed assessment of both short-term and long-term health outcomes for mothers who undergo VBAC and their children, compared with those who have ERCS, within a specific regional population in Saudi Arabia.

**Methods and analysis:**

This retrospective cohort study uses data routinely collected from the BESTCare electronic medical record system managed by the Ministry of National Guard Health Affairs. The eligible participants are women of reproductive age (15–49 years) with a history of one previous CS (either ERCS or emergency CS) followed by a vaginal birth. The study will involve graphical, descriptive and analytical statistics, including multiple logistic regression to assess VBAC outcomes. During the exploratory phase, principal component analysis will identify key domains related to VBAC. Additionally, workshops involving study researchers and both internal and external panels of experts will review interim analyses, allowing for a stepwise assessment of the project’s progress.

**Ethics and dissemination:**

The study received approval from the Institutional Review Board at the King Abdullah International Medical Research Center, with approval number NRR24/089/9, in accordance with the Saudi Law of Ethics of Research on Living Creatures. The findings of this study will be disseminated through publication in a peer-reviewed scientific journal and through dissemination workshop.

**Strengths and limitations of this study**

- The study uses a detailed sub-national electronic medical record system (BESTCare) that keeps digitised, complete and rigorously validated individual records.
- The study will evaluate various short-term and long-term clinical, psychological and cognitive health outcomes to offer a comprehensive view of vaginal birth after caesarean section.
- Procedural or diagnostic misclassification can happen because of differences in physician reviews and clinical documentation processes, which may result in incomplete or missing registry data.
- The gestational age definition in this study does not completely match the updated standards in use during the study period, potentially influencing the definitions of related obstetric terms.

## Introduction

 Globally, rates of caesarean section (CS) have risen significantly over past decades. An estimated 21% of all births were delivered via CS in 2018, with forecasts predicting that nearly one-third (29%) of all births will occur via CS by 2030.^1^ The WHO does not recommend specific CS rates for individual countries or hospitals; however, rates up to 10% are associated with reduced maternal and neonatal mortality. Interestingly, rates above this threshold do not show additional mortality benefits. The WHO emphasises the need to avoid medically unnecessary CSs, which can pose risks to both the mother and the baby. However, efforts should focus on providing CS deliveries to women in need, rather than targeting or achieving a specific rate.^2 3^

Global increases in CS delivery rates are driven by the complex interplay among medical, social and health system factors. Medically, increasing maternal age, higher rates of obesity and the growing use of assistive reproductive technology have all resulted in more pregnancies being classified as ‘high-risk’, and such pregnancies often necessitate a surgical delivery.^4^

Social and cultural influences also play a significant role. Some women opt to undergo a CS because they fear labour pain. Others perceive the procedure as more convenient and easier to integrate into busy daily schedules; societal norms also favour surgical births. In certain countries, CS births are considered to symbolise modernity or wealth.^5 6^ Health system dynamics further reinforce this trend—financial incentives for providers, fear of litigation and declining skills in managing vaginal births have led to a preference for CS in many settings.^4 5 7^

While CS can save both maternal and neonatal lives when medically indicated, its overuse poses significant risks, particularly in settings with limited surgical capacity.^8^ Vaginal birth after CS (VBAC) offers a safe and evidence-based alternative to elective repeat CS (ERCS) deliveries for many women who have undergone a prior CS. VBAC is associated with lower maternal morbidity, shorter recovery times and reduced healthcare costs.^9^

Despite its benefits, VBAC remains underused in many countries due to clinical, institutional and sociocultural uncertainties.^9^ Successful VBAC is associated with reduced surgical risks—including lower rates of infection and haemorrhage, fewer postoperative complications, shorter hospital stays and faster recovery times—compared with repeat CS.^10^ VBAC may also lower the likelihood of placenta accreta and other complications during future pregnancies; these complications are more common in women who undergo ERCS.^10^

Psychologically, many women report greater satisfaction and a stronger sense of empowerment following a successful VBAC^11^; however, VBAC procedures carry their own risks, the most serious being uterine rupture. Despite its rarity (0.3%–1%), uterine rupture can be life-threatening and may necessitate an emergency hysterectomy or emergency CS. Importantly, emergency procedures such as these are associated with higher morbidity than planned procedures. VBAC eligibility depends on factors such as the type of previous uterine incision, the number of prior caesareans and the local availability of emergency obstetric care.^12 13^

Saudi Arabia’s CS rate rose from 19.1% (2008–2011) to 27% in 2016^1 14^ and, according to the Saudi General Authority of Statistics, reached 28.1% in 2024.^15^ The Saudi healthcare model has also recently shifted toward decentralised, insurance-funded care as part of the ongoing national health reform under Saudi Vision 2030.^16^ The Mother-Baby-Family Friendly Maternity Care Facility initiative by the Saudi Arabian Ministry of Health aims to reach a VBAC rate of 75% or more by 2030, with the current rate at 60%. This goal aligns with the Saudi National Ministry of Health’s recommendations for the childbirth initiative.^17^

However, Saudi Arabia lacks the local evidence and guidance necessary to support this goal. A recent Saudi study assessing the awareness and barriers to VBAC among women with a prior CS found that around 63% had received no advice or counselling from their physicians about VBAC as a delivery option.^18^ Despite a rising number of VBAC attempts nationally, the exact prevalence of VBAC in any defined population remains outside official estimates and there are no national-level mechanisms for tracking patterns of VBAC use. Moreover, we have no information on the lack of existing mechanisms for VBAC use patterns at a national level.

A 2018 Saudi study examining VBAC success rates and short-term outcomes found that women attempting VBAC faced a higher risk of conversion to CS due to fetal distress, while a prior normal vaginal delivery significantly increased the chance of successful VBAC.^19^ However, that study focused on a discrete set of short-term outcomes and did not address the crucial longer-term maternal and child clinical, psychosocial or cognitive consequences.^19^ Similarly, a second study to assess the outcome of VBAC with a focus on grand multiparous women in comparison with multiparous women found no statistical significance in postpartum haemorrhage or hospital stay duration.^20^ No Saudi study has comprehensively examined the prevalence and predictors of VBAC or evaluated its short-term and long-term outcomes to guide clinical practice in the Kingdom—a gap that remains a crucial unfinished task on the national health transformation agenda under Saudi Vision 2030.^21^

This study will use available electronic medical data from the Ministry of National Guard Health Affairs (MNGHA) sub-national hub (BESTCare)^22^ to generate evidence to inform policy and programmes on short-term and long-term health and obstetric outcomes of VBAC across different delivery modes among mothers and their children in Saudi Arabia.

## Methods and analysis

### Patient and public involvement

None.

### Study design

This retrospective cohort study uses routinely collected data from the MNGHA sub-national BESTCare system, the study will start from November 2026. We anticipate study completion in November 2028. The BESTCare system has gathered extensive health and non-health-related information since 2016. The data is sourced from hospital records at the MNGHA facility in Riyadh, Saudi Arabia.^23^ Using BESTCare’s standardised, high-quality clinical records—such as electronic medical records (EMRs), birth registries and maternal and paediatric logs—enables thorough analyses unaffected by recall bias and facilitates precise monitoring of important outcomes.^22^ The BESTCare hub contains maternal EMRs, detailed birth profiles, follow-up records for mothers and children, and clinical and administrative data. This setup offers comprehensive, long-term data crucial for research on maternal and child health outcomes. A list of specific indicators can be found in online supplemental file 1.

### Research question

What are the short-term and long-term clinical, psychosocial and cognitive outcomes of VBAC across different delivery modes for women with a prior CS delivery in Saudi Arabia from 2019 to 2024?

This study aims to achieve two main objectives:

1. To estimate the prevalence of VBAC within a sub-national Saudi population, using hospital records from the MNGHA hospital in Riyadh.
2. To provide a comprehensive comparison of short-term and long-term health outcomes for mothers and children who undergo VBAC vs those who have an ERCS.

‘Short-term maternal outcomes’ refer to health conditions that appear during the postpartum period, up to 6 weeks after childbirth, a vital time for identifying and addressing complications. Conversely, ‘long-term maternal outcomes’ involve health issues that develop after 6 weeks and up to 12 months postpartum. These may include ongoing physical or mental health problems and can affect future pregnancies, fertility or lead to chronic health conditions.^24^ Therefore, maternal outcomes were categorised into short-term and long-term groups according to their definitions, as outlined in the literature.^25^ The child’s short-term outcomes are their physical, mental or developmental health observed within the first 3 years after VBAC; outcomes beyond this period will be considered long-term.^25^ A defined list of child outcomes and their International Statistical Classification of Diseases and Related Health Problems, 10th Revision (ICD-10) codes is provided in online supplemental file 2.

1. To identify the main domains linked to VBAC outcomes by streamlining the data structure and the subdomains (cognitive, psychosocial and clinical outcomes) that influence VBAC results.
2. To examine the potential health system and social factors influencing VBAC health outcomes.

### Study population and sampling

All eligible pregnancies documented in the BESTCare hub from 2019 onwards will be included. This hub guarantees comprehensive recording of all cases that meet the inclusion criteria, eliminating the need for a pre-specified sample size and thereby improving statistical power and the generalisability of the findings.^26 27^

### Inclusion criteria

Eligible participants will be women of reproductive age (15–49 years), with histories of prior CS (ERCS or emergency), followed by a vaginal birth.^28^ All previous CS procedures will be recorded, regardless of the outcome, associated complications or birth status. The vaginal birth that occurs following the CS may be spontaneous or assisted (eg, vacuum extraction or forceps delivery).

Participants will be identified based on their assigned ICD-10 codes,^29^ particularly O34.21 (maternal care for scar from previous CS delivery). Included participants will also carry at least one surgical procedure (code) that indicates a vaginal birth. Neonates delivered via VBAC during the same period will also be included. To ensure comprehensive outcome capture and appropriate comparators, the VBAC group will include both successful VBACs and Trial of Labor After Cesarean (TOLAC), defined as ‘an elective planned attempt by women with previous caesarean delivery to achieve vaginal birth that ended in repeat CS’.

### Exclusion criteria

Women with multiple previous CSs and no VBAC, those who delivered only vaginally in all pregnancies, women whose VBAC attempts happened outside of Saudi MNGHA hospitals, or women who had vaginal birth before their CS—since they do not qualify as a VBAC trial group—will be excluded.

### Follow-up modality and outcome surveillance

All eligible women are monitored over time through regular antenatal and postnatal visits, recorded in the BESTCare system. Follow-up usually starts in the first trimester and aligns with the Saudi Ministry of Health’s national maternal care guidelines. Pregnant women are typically monitored every month until 28 weeks of gestation, then every 2 weeks until 36 weeks and weekly after that. Postpartum care involves hospital follow-up for up to 6 weeks, with extended data tracked through the Integrated Care Model across MNGHA hospitals to assess long-term maternal health outcomes. This data collection encompasses both clinical and non-clinical information:

- Maternal complications include haemorrhage, uterine rupture and infections. A comprehensive list of maternal outcomes is available in online supplemental file 2.
- Neonatal parameters include the Apgar score (appearance, pulse, grimace, activity, respiration), birth weight, neonatal intensive care unit (NICU) admission and the child’s health outcomes such as asthma, diabetes, autism and neurodevelopmental status, especially at the 2-year paediatric milestones. A detailed list of child outcomes is available in online supplemental file 3.

Infants and children have routine follow-up visits at 2, 4, 6, 9, 12, 18 and 24 months, followed by annual check-ups. Longer-term maternal outcomes beyond 3 years postpartum include issues such as urinary or faecal incontinence, impaired fertility, mental health conditions and chronic diseases. All data are systematically collected from BESTCare EMRs, based on predefined criteria and case definitions, encompassing sociodemographic information, diagnoses, medications, laboratory results and hospital events. Paediatric follow-up through the MNGHA child health programme provides continuous health monitoring for children.

### Clinical management of VBAC in Saudi Arabia

In Saudi Arabia, women with one prior CS who are eligible for a trial of TOLAC receive standard antenatal care throughout pregnancy. Starting at 36 weeks, they get personalised counselling about their delivery options. Obstetricians and midwives discuss the potential advantages and risks of both TOLAC and ERCS, helping women make informed choices. Those opting for TOLAC must give written informed consent before labour and must still meet criteria for TOLAC. Labour induction may be performed using mechanical methods in accordance with local hospital protocols. During labour, women attempting VBAC are continuously monitored with electronic fetal monitoring from active labour until delivery to detect fetal issues early and ensure safety. Care during labour involves a multidisciplinary team: obstetricians manage women with higher risks. At the same time, nurses provide continuous bedside care and alert obstetricians to any concerns. This teamwork aims to ensure the safety of both mother and baby.

Ultimately, the woman’s decision determines the delivery mode after thorough counselling. When there are no contraindications, obstetricians usually advise eligible women to consider TOLAC, as a successful VBAC can reduce the rate of repeat CSs and their associated short-term and long-term risks.

### Data collection

Delivery characteristics will cover the mode of delivery (such as VBAC, instrumental vaginal delivery with vacuum or forceps, elective CS or emergency CS), along with labour induction, intrapartum complications and birth order.^30^

Maternal demographic and behavioural variables include age, nationality, education level, socioeconomic status, smoking history, physical activity and dietary habits.^31^

Maternal outcomes encompass both immediate postpartum and longer-term effects. Immediate outcomes include postpartum haemorrhage, uterine rupture, hysterectomy, severe infection, blood transfusion and maternal death. Psychological outcomes like postpartum depression and anxiety are evaluated during the first antenatal visit as part of routine maternity care, aligned with the Pregnancy and Childbirth International Consortium for Health Outcomes Measurement (ICHOM). Data on postpartum depression and anxiety will be gathered from clinical assessments documented at this visit, while pre-existing mental health conditions recorded before or during pregnancy will be obtained from the electronic pregnancy diary using the Edinburgh Postnatal Depression Scale.^32^ These variables will serve as covariates in the analyses to control their potential impact on postpartum psychological health.

Obstetric and medical history variables include body mass index, gravidity, parity, interpregnancy interval, type and indication of prior CS (elective or emergency), antenatal care attendance and maternal comorbidities such as gestational diabetes, preeclampsia and chronic hypertension.^33^

Long-term outcomes (assessed at 6 and 12 months postpartum) will include urinary and faecal incontinence, chronic pelvic pain, infertility, diabetes mellitus and psychological sequelae, where available. A list of maternal outcomes is provided in online supplemental file 3.

Neonatal outcomes will be assessed immediately after birth, including gestational age at birth, birth weight, Apgar scores, perinatal mortality (stillbirth or early neonatal death), birth asphyxia, admission to NICU, neonatal infections, massive pulmonary haemorrhage, clavicle fractures, intracranial haemorrhage and meconium aspiration syndrome.

Long-term child outcomes include conditions such as cerebral palsy, asthma, autism spectrum disorder, attention-deficit hyperactivity disorder, obesity and type 1 diabetes mellitus, as recorded in the child’s health follow-up logs during paediatric visits at 3 years of age. When available, cognitive and psychosocial outcomes will be evaluated using standardised developmental screening tools administered during routine check-ups. Cognitive outcomes will also be reviewed through physicians’ analysis of clinical notes in electronic health records. Data on cognitive concerns, developmental delays, impairments and all documented cognitive findings will be collected. All variables and outcomes will adhere to the operational definitions outlined in the study’s outcome classification tables (online supplemental files). These standardised definitions are intended to promote data consistency, validity and reproducibility across all datasets.

### Data management and data quality

To maintain high data quality, multiple strategies will be used at each data extraction stage. First, the BESTCare system’s filters will be used to identify all patients with the relevant ICD codes for ‘previous CS delivery’, including indicators of VBAC attempts and outcomes. This system ensures precise patient identification and reliable linkage of maternal and neonatal data.

To improve data accuracy and consistency, abstractors will participate in standardised training sessions led by a team of obstetrician-gynaecologists (OB-GYN), a midwife and statisticians. These will occur at the start of the study and at scheduled intervals during data collection. The training will cover clinical definitions of outcomes, entry procedures and proper interpretation of both structured and unstructured fields in EMRs.^34^ The authors of this protocol are experienced OB-GYNs, paediatric consultants and midwives who oversee data extraction, review all records and ensure compliance with inclusion/exclusion criteria. The data collection process will implement a three-phase quality assurance cycle, as below:

1. Initial collection and panel review
  An initial sample will be assessed by a panel of external, multidisciplinary experts from various Saudi hospitals who are independent of the project. Their task is to resolve definitional ambiguities and assess the risk of misclassification. For example, complex cases with ICD codes that do not match the documented clinical diagnosis will be double-checked by reviewing the delivery logbook and verifying the accuracy of outcome coding. Any cases that seem unlikely or inconsistent will be flagged and discussed to refine operational definitions. Cases that remain ambiguous will be excluded from the analysis.
2. During the initial interim review
  This section will generate descriptive statistics to detect initial patterns and ensure plausibility of the data. It will also verify that the study comprehensively covers the full spectrum of maternal and child outcomes as planned.
3. Full-scale data collection and second interim analysis
  A thorough extraction will be conducted, followed by a second interim analysis to verify the validity of the dataset. Any remaining discrepancies will be resolved through consensus revalidation by the expert panel.

The data management procedures will align with international recommendations for observational study quality, namely, the Strengthening the Reporting of Observational Studies in Epidemiology (STROBE) guidelines.^35^

### Statistical analysis

Maternal and child outcomes will be evaluated and compared across four categories of delivery methods.

1. Vaginal delivery without instruments.
2. Instrumental vaginal delivery (forceps or vacuum extraction).
3. VBAC after emergency CS.
4. VBAC after elective CS.

Quantitative variables like maternal age, gestational age and birth weight will be summarised using means and SD for data that follows a normal distribution or medians and IQRs for data that is skewed. Categorical variables such as mode of delivery and NICU admission will be presented through frequencies, percentages as well as visual and descriptive statistics.

Bivariate analyses will use Pearson’s χ^2^ test (or Fisher’s exact test if appropriate) for categorical data. For continuous variables, independent-samples t-tests or one-way analysis of variance (ANOVA) will be used based on the number of groups and normality assumptions. If these assumptions are violated, non-parametric tests such as the Mann-Whitney U test or the Kruskal-Wallis test will be applied.^36^ To assess the determinants of VBAC, multiple logistic regression models will be used (eg, VBAC and non-VBAC). Predictor variables in the models will be identified using a directed acyclic graph approach to explore potential confounders, effect modifiers or mediators between VBAC and other predictors.^37^ To identify new core domains of maternal and child health outcomes (objective 3), principal component analysis (PCA) will be used during the exploratory phase. PCA will assist in pinpointing the most informative variable combinations and reducing the dimensionality of predictor sets, particularly for models involving long-term child outcomes and developmental diagnoses.

All analyses will be performed using the validated statistical software package STATA (V.19).^38^ All statistical tests will be conducted as two-tailed tests, considering p values <0.05 statistically significant. To assess model fit and validity, diagnostics like the Hosmer-Lemeshow test for logistic regression and residual plots for linear models will be used.

### Interim analysis

An interim analysis will be performed after the initial data collection to assess the completeness, consistency and representativeness of the dataset. This review will take place once 500 records are abstracted and entered. The results will guide any needed adjustments to data abstraction processes or model setup.

### Ethics and dissemination

This study received approval from the Institutional Review Board (IRB) at King Abdullah International Medical Research Center (KAIMRC), with approval number NRR24/089/9. It was conducted in accordance with the Saudi Law of Ethics of Research on Living Creatures (2010) and adhered to international ethical standards, including the Declaration of Helsinki and Council for International Organizations of Medical Sciences guidelines.^39^

Data will be securely stored in controlled-access institutional systems, with encrypted backups maintained in accordance with KAIMRC and IRB ethical standards. Two workshops will review the study findings from interim and final analyses and assist in developing clinical guidelines based on the results. The project’s outcomes will be disseminated through multiple publications, including one on maternal short-term and long-term outcomes and another on child outcomes over the same periods. To ensure privacy and confidentiality, all personally identifiable information will be removed during data extraction. Additional encrypted, password-protected backups will be stored on authorised servers in accordance with MNGHA’s data governance framework. In line with MNGHA and KAIMRC IRB policies, all data and study materials will be securely kept for at least 5 years after the study ends, supporting future audits or secondary research. The findings of this study will be disseminated through publication in a peer-reviewed scientific journal and through dissemination workshop, the workshop will involve relevant members from the Saudi medical board, hospital stakeholders, physicians and midwives.

### Monitoring of adverse events

This study relies on EMRs and existing data. Any adverse events or ethical concerns that arise during analysis will be promptly reported to the IRB in accordance with the approved protocol. The IRB will continue its monitoring throughout data management and dissemination.

### Participant welfare and non-coercion

Since the study does not involve direct contact with participants, it poses minimal risk. No financial rewards or coercion will be used. All collected data will be used solely for research purposes aimed at enhancing maternal and neonatal health services in Saudi Arabia.

### Data availability statement

Upon completion of the study, the data generated and/or analyzed will be available from the corresponding author upon request.

## Supplementary material

10.1136/bmjopen-2025-114754 online supplemental file 1

10.1136/bmjopen-2025-114754 online supplemental file 2

10.1136/bmjopen-2025-114754 online supplemental file 3

## References

[1] Betran AP, Ye J, Moller A-B (2021). Trends and projections of caesarean section rates: global and regional estimates. BMJ Glob Health.

[2] World Health Organization (2018). WHO recommendations: non-clinical interventions to reduce unnecessary caesarean sections. https://www.who.int/publications/i/item/9789241550338.

[3] World Health Organization (2015). WHO statement on caesarean section rates. https://www.who.int/publications/i/item/WHO-RHR-15.02.

[4] Pavlidou E, Antasouras G, Papadopoulou SK (2023). Association of Maternal Risk Factors with the Prevalence of Caesarean Section Deliveries: A Cross-Sectional Study. Med Sci (Basel).

[5] Panda S, Daly D, Begley C (2018). Factors influencing decision-making for caesarean section in Sweden – a qualitative study. BMC Pregnancy Childbirth.

[6] Alipour A, Hantoushzadeh S, Hessami K (2022). A global study of the association of cesarean rate and the role of socioeconomic status in neonatal mortality rate in the current century. BMC Pregnancy Childbirth.

[7] Yu S, Fiebig DG, Viney R (2022). Private provider incentives in health care: The case of caesarean births. Soc Sci Med.

[8] Kingdon C, Downe S, Betran AP (2018). Non-clinical interventions to reduce unnecessary caesarean section targeted at organisations, facilities and systems: Systematic review of qualitative studies. PLoS One.

[9] Barnea ER, Ramasauskaite D, Ubom AE (2025). FIGO good practice recommendations for vaginal birth after cesarean section. Int J Gynecol Obstet.

[10] American College of Obstetricians and Gynecologists, Society for Maternal-Fetal Medicine (2018). Obstetric Care Consensus No. 7: Placenta Accreta Spectrum. Obstet Gynecol.

[11] Olza I, Leahy-Warren P, Benyamini Y (2018). Women’s psychological experiences of physiological childbirth: a meta-synthesis. BMJ Open.

[12] Didier-Mathon H, Kayem G, Thubert T (2025). Risk factors for complete uterine rupture in patients with trial of labor after cesarean delivery. Acta Obstet Gynecol Scand.

[13] Qiu L, Zhu J, Lu X (2023). The safety of trial of labor after cesarean section (TOLAC) versus elective repeat cesarean section (ERCS): a systematic review and meta-analysis. J Matern Fetal Neonatal Med.

[14] Ahmed AE, Mohammad RS (2018). Cesarean sections. Associated factors and frequency at King Abdulaziz Medical City in the Central Region of the Kingdom of Saudi Arabia. Saudi Med J.

[15] General Authority for Statistics (2024). Reproductive Care Statistics in the Kingdom 2024. https://www.stats.gov.sa/documents/20117/2435273/Women+Health+and+Reproductive+Care+Statistics+Publication+2024+EN.pdf/33569118-478b-3be4-a6ef-d291a8399d11?t=1735117033009.

[16] Hazazi A, Wilson A, Larkin S (2022). Reform of the Health Insurance Funding Model to Improve the Care of Noncommunicable Diseases Patients in Saudi Arabia. Health Care (Don Mills).

[17] Saudi Arabia National Ministry of Health (2023). Mother Baby-Family Friendly Maternity Care Facility (MBFMF) Initiative Guidebook.

[18] Aloufi D, Saggaf O, Alkhalifah ZA (2025). Exploring barriers and facilitators of vaginal birth after caesarean section (VBAC), awareness, and preferences among females in Western Saudi Arabia. J Family Med Prim Care.

[19] Kiwan R, Al Qahtani N (2018). Outcome of vaginal birth after cesarean section: A retrospective comparative analysis of spontaneous versus induced labor in women with one previous cesarean section. Ann Afr Med.

[20] Yamani Zamzami TY (2004). Vaginal birth after cesarean section in grand multiparous women. Arch Gynecol Obstet.

[21] Chowdhury S, Mok D, Leenen L (2021). Transformation of health care and the new model of care in Saudi Arabia: Kingdom’s Vision 2030. J Med Life.

[22] Aldosari B, Alanazi A (2018). Documentation integrity: Authorship functionalities of EHR in a Saudi Arabian hospital. Comput Biol Med.

[23] (2025). Ministry of National Guard Health Affaris (MNGHA). https://ngha.med.sa/english/AboutNGHA/Pages/default.aspx.

[24] (2019). ACOG Practice Bulletin no.205: Vaginal Birth After Cesarean Delivery. https://journals.lww.com/greenjournal/abstract/2019/02000/acog_practice_bulletin_no__205__vaginal_birth.40.aspx.

[25] Sandall J, Tribe RM, Avery L (2018). Short-term and long-term effects of caesarean section on the health of women and children. Lancet.

[26] Al-Rifai RH, Ali N, Barigye ET (2020). Maternal and birth cohort studies in the Gulf Cooperation Council countries: a systematic review and meta-analysis. Syst Rev.

[27] Say L, Barreix M, Chou D (2016). Maternal morbidity measurement tool pilot: study protocol. Reprod Health.

[28] World Helath Organization (2025). Women of reproductive age. https://www.who.int/data/gho/indicator-metadata-registry/imr-details/women-of-reproductive-age-%2815-49-years%29-population-%28thousands%29?utm_source=chatgpt.com.

[29] Hernandez-Ibarburu G, Perez-Rey D, Alonso-Oset E (2019). ICD-10-PCS extension with ICD-9 procedure codes to support integrated access to clinical legacy data. Int J Med Inform.

[30] Habib F, Faidh R, Aljohani S (2020). A retrospective study about the outcome of vaginal delivery after cesarean section in Madinah, Saudi Arabia. IJMDC.

[31] Fayed AA, Wahabi H, Mamdouh H (2017). Demographic profile and pregnancy outcomes of adolescents and older mothers in Saudi Arabia: analysis from Riyadh Mother (RAHMA) and Baby cohort study. BMJ Open.

[32] Cox JL, Holden JM, Sagovsky R (1987). Detection of postnatal depression. Development of the 10-item Edinburgh Postnatal Depression Scale. Br J Psychiatry.

[33] Sung S, Mikes BA, Martingano DJ (2025). Cesarean Delivery.

[34] Boyle CA, Boulet S, Schieve LA (2011). Trends in the prevalence of developmental disabilities in US children, 1997-2008. Pediatrics.

[35] von Elm E, Altman DG, Egger M (2007). The Strengthening the Reporting of Observational Studies in Epidemiology (STROBE) statement: guidelines for reporting observational studies. Prev Med.

[36] Kenneth Rothman J (2008). Modern Epidemiology.

[37] Babyak MA (2004). What you see may not be what you get: a brief, nontechnical introduction to overfitting in regression-type models. Psychosom Med.

[38] STATA Program (2025). The complete statistical software for data science. https://www.stata.com/.

[39] M RDN (2017). Law of Ethics of Research on Living Things Law of Ethics of Research on Living Creatures.

