# Supplementary material for: Clinical, psychosocial and cognitive outcomes following VBAC: a protocol for a sub-national cohort study in Saudi Arabia (2019–2024)
Source: BMJ Open. 2026 Oct 13;16(10):e114754. doi: 10.1136/bmjopen-2025-114754 (PMC13663696; doi:10.1136/bmjopen-2025-114754)
Supplement: Supplementary file 1 [file bmjopen-2025-114754.reviewer_comments.pdf]

PEER REVIEW HISTORY

BMJ Open publishes all reviews undertaken for accepted manuscripts. Reviewers are asked to complete a checklist review form and are provided with free text boxes to elaborate on their assessment. These free text comments are reproduced below.

ARTICLE DETAILS

Title (Provisional)

Clinical, psychosocial, and cognitive outcomes following VBAC: a protocol for a subnational cohort study in Saudi Arabia (2019-2024)

Authors

Aldawsari, Asma A; Alzomia, Sufyan; Alanizi, Nouf F; Thomas, Seena; Paras, Bienvenido; Alnasser, Lubna A; Alghamdi, Abdullah; Dagestani, Hattan F; Moller, Ann-Beth; Hussain-Alkhateeb, Laith

---

VERSION 1 - REVIEW

**Reviewer**	**1**
**Name**	**Doherty, Anne**
**Affiliation**	**University College Dublin, Department of Psychiatry**
**Date**	**10-Apr-2026**
**COI**	**None**

Many thanks for this interesting and well-timed proposal on an important topic. The rationale is clearly outlined with appropriate reference to both the literature and considering the local context too. The methodology is clear, and appears to be appropriate.

There is really just one question from me - in the title the outcomes are described across 3 domains "Clinical, psychosocial, and cognitive".

However it appears from the methodology that there will not be any cognitive data collected. The only outcome that could be considered 'psychosocial' is "Psychological disorder (postpartum depression/anxiety)". so

a) unless there are more 'psychosocial, and cognitive' outcomes which are not listed, the title may need to be revised

b) how will "Psychological disorder (postpartum depression/anxiety)" be assessed. Are scales such as the EPDS being used, is it based on any form of assessment? Or is it based on referral to mental health services (and are there integrated perinatal mental health services in SA)? Will pre-existing mental health problems be considered (and how will they be treated in the analysis)

**Reviewer**	**2**
**Name**	**Feeley, Claire**
**Affiliation**	**King's College London**
**Date**	**05-Jun-2026**
**COI**	**None**

Thank you for giving me the opportunity to review this protocol exploring the clinical, psychosocial, and cognitive outcomes following VBAC: a protocol for a subnational study in Saudi Arabia. It is vital that we have different country context data and I welcome this study! Some minor comments below:

Abstract:

Intro-This point 'Despite the recognized benefits of VBAC, there remains a critical lack of robust evidence linking VBAC practices to potential clinical, psychosocial, and cognitive health outcomes for both mothers and their children' – I don't feel this is fully substantiated within the global evidence base, needs further consideration. Perhaps you mean in the Saudi context?

Methods and analysis - Is this retrospective or prospective data collection? Or both? Please clarify.

Introduction:

Points on lines 84-87, 101-104, need evidence to support these statements.

127-131 – missing reference, it is an important study.

How is VBAC usually managed in Saudi e.g., is spontaneous labour encouraged/awaited for and if induction is ever used, what types of induction? What type of fetal monitoring is used for VBAC? Additionally, who facilitates intrapartum care in Saudi context, is it midwives or obstetricians or a mixture? These care practices would influence outcomes and should be explained to help understand the Saudi context.

Methods:

Is this retrospective or prospective or both? Please state.

194-199 I am not following your eligibility criteria, reads as quite confusing. Could you please rephrase. (The exclusion criteria are clear and can follow that well).

VERSION 1 - AUTHOR RESPONSE

We would like to thank the Editor and Reviewers for their valuable comments and suggestions. We have carefully considered all comments and revised the manuscript accordingly. Our point-by-point responses are provided below.

Reviewer Comment	Author Response	Location in Revised Manuscript
Reviewer 1, Comment 1: There is really just one question from me - in the title the outcomes are described across 3 domains "Clinical, psychosocial, and cognitive". However it appears from the methodology that there will not be any cognitive data collected. The only outcome that could be considered 'psychosocial' is "Psychological disorder (postpartum depression/anxiety)". so a) unless there are more 'psychosocial, and cognitive' outcomes which are not listed, the title may need to be revised.	Thank you for this valuable comment. We appreciate the opportunity to clarify this point. Cognitive outcomes will indeed be assessed in the study; however, information on cognitive outcomes will be extracted from physicians' clinical notes documented in the electronic health records. To address the reviewer's concern, we have revised the methods section to explicitly describe the collection and assessment of cognitive outcomes from physicians and nurses' documentation.	Page 15. Lines 298, 299, 300.
Reviewer 1, Comment 2: b) how will "Psychological disorder (postpartum depression/anxiety)" be assessed. Are scales such as the EPDS being used, is it based on any form of assessment? Or is it based on referral to mental health services (and are there integrated perinatal mental health services in SA)? Will pre-existing mental health problems be considered (and how will they be treated in the analysis)	Thank you for this important comment. Psychological outcomes will be assessed as part of the routine maternal care pathway in accordance with the International Consortium for Health Outcome Measures (ICHOM)Pregnancy and Childbirth Standard Set. Women undergo routine mental health assessment during their first antenatal visit, and postpartum psychological assessment is included as part of the planned follow-up care using EPDS form. Information on postpartum depression and anxiety will be obtained from the electronic health records based on these documented clinical assessments. In addition, pre-existing mental health conditions identified during the antenatal assessment or documented in the medical records will be extracted and considered in the statistical analysis.	Page 14. Lines: 271-279.
Reviewer 2, Comment 1: Abstract: Intro-This point 'Despite the recognized benefits of VBAC, there remains a critical lack of robust evidence linking VBAC practices to potential clinical, psychosocial, and cognitive health outcomes for both mothers and their children' – I don't feel this is fully substantiated within the global evidence base, needs further consideration. Perhaps you mean in the Saudi context?	Thank you for this important comment. We agree that the original statement may have overstated the evidence gap at the global level, where a growing body of literature has evaluated VBAC-related outcomes. Our intention was to highlight the limited evidence available within the Saudi Arabian context. Accordingly, we have revised the statement to clarify that the evidence gap pertains primarily to Saudi Arabia and similar regional settings, where comprehensive data on maternal and child outcomes following VBAC remain scarce. This revision improves the	Page 3. Lines 38-39.

	accuracy of the statement and better reflects the rationale for conducting the present study.	
Reviewer 2, Comment 1: Methods and analysis - Is this retrospective or prospective data collection? Or both? Please clarify.	Thank you for this comment. We agree that the study design was not sufficiently clear in the original manuscript. We have revised the Methods section to explicitly state that this study employs a retrospective cohort design, utilizing routinely collected data from electronic health records and national registries. No prospective data collection will be undertaken.	Page 8. line: 152
Reviewer 2, Comment 2:Introduction: Points on lines 84-87, 101-104, need evidence to support these statements. 127-131 – missing reference, it is an important study.	The required references have been added to support the statements in the indicated sections.	Page 5. Lines: 86-89.<br>Page 6. Lines: 103-105<br>Page 7. Lines: 129-133
Reviewer 2, Comment 3: How is VBAC usually managed in Saudi e.g., is spontaneous labour encouraged/awaited for and if induction is ever used, what types of induction? What type of fetal monitoring is used for VBAC? Additionally, who facilitates intrapartum care in Saudi context, is it midwives or obstetricians or a mixture? These care practices would influence outcomes and should be explained to help understand the Saudi context.	Thank you for this valuable comment. A new detailed subsection entitled "Clinical Management of VBAC" has been added to the protocol.	Page 12,13. Lines: 241-261
Reviewer 2, Comment 4: Methods:<br>Is this retrospective or prospective or both? Please state.	Thank you for this comment. We agree that the study design was not sufficiently clear in the original manuscript. We have revised the Methods section to explicitly state that this study employs a retrospective cohort design, utilizing routinely collected data from electronic health records and national registries. No prospective data collection will be undertaken.	Page 8. line: 152
Reviewer 2, Comment 5: 194-199 I am not following your eligibility criteria, reads as quite confusing. Could you please rephrase? (The exclusion criteria are clear and can follow that well).	Thank you for this helpful comment. To improve readability and ensure consistency with the exclusion criteria, we have revised this section to provide a clearer description of the study population and eligibility requirements.	Page 10. Lines: 196-203

VERSION 2 - REVIEW

Reviewer 2

**Name**	**Feeley, Claire**
**Affiliation**	**King's College London**
**Date**	**10-Aug-2026**
**COI**	

Thank you for giving me the opportunity to re-review this protocol submission, the authors have adequately addressed my comments and I look forward to seeing the results of this study!
