# Supplementary material for: Clinical, psychosocial and cognitive outcomes following VBAC: a protocol for a sub-national cohort study in Saudi Arabia (2019–2024)
Source: BMJ Open. 2026 Oct 13;16(10):e114754. doi: 10.1136/bmjopen-2025-114754 (PMC13663696; doi:10.1136/bmjopen-2025-114754)
Supplement: Supplementary file 2 [file bmjopen-16-10-s001.docx]

Supplementary file 1. Detailed indicators and maternal comorbidities

### A. Demographic and social indicators

-Maternal age (years)

-Nationality

-Education level

-Occupation/employment status

-Marital status

-Socioeconomic status/insurance

-Residence (city/region)

-Gravidity, Parity

-Interpregnancy interval (months)

-Pre‑pregnancy BMI; height/weight at booking (BMI: kg/m²)

-Gestational age at booking visit

### B. Maternal comorbidities

-Chronic hypertension

-Pre‑gestational diabetes (Type 1/Type 2)

-Gestational diabetes (A1/A2)

-Preeclampsia / HELLP syndrome (if antepartum)

-Thyroid disease (hypo/hyper)

-Asthma / chronic lung disease

-Renal disease / chronic kidney disease (CKD)

-Autoimmune disease (e.g., systemic lupus erythematosus [SLE], multiple sclerosis [MS])

-Cardiac disease

-Hematologic disorders (e.g., thrombophilia)

-Smoking or nicotine exposure during pregnancy

-Other significant comorbidity (specify)

### C. Pregnancy and intrapartum indicators

-Antenatal complications (placenta previa / accreta suspicion, abruption)

-Onset of labor (spontaneous/induction/no labor – ERCD)

-Induction or augmentation method (oxytocin, prostaglandin)

-Analgesia or anesthesia (epidural/spinal/general)

-Mode of delivery (VBAC, instrumental vaginal, emergency CS, elective CS)

-Duration of labor (first/second stage if available)

-Intrapartum fever or chorioamnionitis

-Shoulder dystocia/operative vaginal delivery

-Estimated blood loss (mL)

### D. Short-Term Maternal Outcomes (Delivery to ≤6 Weeks)

-Postpartum hemorrhage (PPH) – cumulative blood loss ≥1000 mL *or* blood loss with clinical signs/symptoms of hypovolemia within 24 hours postpartum.

* *Definition:* postpartum hemorrhage (PPH) follows the definition of the American College of Obstetricians and Gynecologists (ACOG): cumulative blood loss ≥1000 mL, or blood loss accompanied by signs or symptoms of hypovolemia, within 24 hours after birth. (1)

-Blood transfusion (units/products)

-Surgical injury (bladder/bowel/ureter)

-Uterine rupture or dehiscence

-Uterine atony

-Peripartum hysterectomy

-Endometritis/wound infection

-Thromboembolism (PE/DVT)

-ICU or HDU admission; length of stay (days)

-Anesthesia-related complications

-Postoperative analgesia requirement (opioid given yes/no)

-Sepsis during labor or postpartum

-Obstetric shock

-Acute renal failure (postpartum)

-Fever >38.5 °C (intrapartum/puerperal)

-Urinary tract infection (cystitis/pyelonephritis)

-Urinary retention

-Wound dehiscence/infection

-Breastfeeding complications

-Puerperal mental disorder (acute onset)

-Readmission ≤6 weeks; reason (coded)

-Maternal death ≤42 days postpartum

### E. Long-Term Maternal Outcomes (>6 Weeks to 12 Months)

-Urinary incontinence

-Anal incontinence

-Uterovaginal prolapse

-Chronic pelvic pain or CS-scar pain

-Adhesions/ileus/subileus

-Caesarean scar hernia

-Psychological disorder (postpartum depression/anxiety)

-Hypertension (new or persistent) – Defined as systolic blood pressure ≥140 mmHg and/or diastolic blood pressure ≥90 mmHg on at least two measurements taken ≥4 hours apart, OR continued requirement for antihypertensive medication beyond 6 weeks postpartum. (2)

-Type 1 or Type 2 diabetes (post-pregnancy onset)

-Auto-immune disease (rheumatoid arthritis [RA], systemic lupus erythematosus [SLE], multiple sclerosis [MS])

-Placenta previa in subsequent pregnancy

-Placenta accreta spectrum (accreta/increta/percreta)

-Uterine rupture in subsequent pregnancy

-Ectopic pregnancy (subsequent conception)

-Miscarriage (subsequent conception)

-Stillbirth (subsequent pregnancy)

-Infertility after index pregnancy

-Peripartum hysterectomy (subsequent pregnancy)

-Abnormal placentation care pathway (planned multidisciplinary management)

-Chronic pelvic adhesions documented post-CS

-Maternal death ≥1 year postpartum (obstetric sequelae)

### References

*1.American College of Obstetricians and Gynecologists.* ***Practice Bulletin No. 183: Postpartum Hemorrhage.*** *Obstet Gynecol. 2017;130(4):e168–e186.* <https://journals.lww.com/greenjournal/abstract/2017/10000/practice_bulletin_no__183__postpartum_hemorrhage.56.aspx>

*2. American College of Obstetricians and Gynecologists. ACOG Practice Bulletin No. 222: Gestational Hypertension and Preeclampsia. Obstetrics & Gynecology. 2020;135(6):e237–e260.* <https://www.acog.org/clinical/clinical-guidance/practice-bulletin/articles/2020/06/gestational-hypertension-and-preeclampsia>
