# Supplementary material for: Clinical, psychosocial and cognitive outcomes following VBAC: a protocol for a sub-national cohort study in Saudi Arabia (2019–2024)
Source: BMJ Open. 2026 Oct 13;16(10):e114754. doi: 10.1136/bmjopen-2025-114754 (PMC13663696; doi:10.1136/bmjopen-2025-114754)
Supplement: Supplementary file 3 [file bmjopen-16-10-s002.docx]

**Supplementary file 2.Maternal outcomes and ICD-10 codes**

## Section A: Maternal Comorbidities

| **Specific Condition** | **ICD-10 Code** | **Notes (Meaning + VBAC Relevance)** |
| --- | --- | --- |
| Chronic hypertension | O10 | Pre-existing hypertension complicating pregnancy — key baseline risk for preeclampsia, placental complications, and VBAC morbidity. |
| Pre-gestational diabetes  (Type 1/2) | O24 | Pre-existing diabetes complicating pregnancy — linked to fetal macrosomia and increased likelihood of repeat cesarean. |
| Gestational diabetes | O24 | Diabetes arising in pregnancy — associated with induction, fetal size, and lower VBAC success rates. |
| Preeclampsia/ HELLP syndrome | O14 | Hypertensive disorder of pregnancy — major contributor to maternal morbidity and contraindication for VBAC in severe forms. |
| Thyroid disease (hypo/hyper) | O99* | Endocrine disease complicating pregnancy — affects metabolism and pregnancy outcomes; relevant for postpartum follow-up. |
| Asthma/chronic lung disease | O99* | Respiratory condition complicating pregnancy — may influence anesthesia management and intrapartum care. |
| Renal disease/CKD | O99* | Renal or circulatory disorder complicating pregnancy — associated with hypertension and impaired uterine perfusion. |
| Autoimmune disease (e.g., SLE/ APS) | O99* | Systemic autoimmune disorder complicating pregnancy — may increase risk for thrombosis, preterm birth, and CS. |
| Cardiac disease | O99* | Cardiovascular disease complicating pregnancy — influences anesthesia, fluid balance, and delivery route decision-making. |
| Hematologic disorders (e.g., thrombophilia) | O99* | Blood/coagulation disorder — raises risk of thromboembolism, relevant to peripartum management and postpartum recovery. |
| Smoking / nicotine exposure | O99* | Substance use complicating pregnancy — increases placental complications and poor wound healing post-CS. |
| Other comorbidity | O99* | Other maternal disease complicating pregnancy — placeholder for rare or multi-system conditions affecting VBAC safety. |

* This category includes conditions which complicate the pregnant state, are aggravated by the pregnancy or are a main reason for obstetric care and for which the Alphabetical Index does not indicate a specific rubric in Chapter XV.

## Section B: Pregnancy and Intrapartum Indicators

| **Specific Condition** | **ICD-10 Code** | **Notes (Meaning and VBAC Relevance)** |
| --- | --- | --- |
| Placenta spectrum (accreta) | O44 | Placental disorders including accreta — key determinant for surgical risk and contraindication for VBAC. |
| Placenta accreta spectrum (increta) | O43 | Placental disorders including increta— key determinant for surgical risk and contraindication for VBAC. |
| Placenta accreta spectrum (abruption) | O45 | Placental disorders including abruption — key determinant for surgical risk and contraindication for VBAC. |
| Onset of labor (spontaneous) | O60 | Preterm labor (delivered before 37 weeks). |
| Onset of labor (no labor – ERCD) | O75 | Other complications or variations in labor onset or delivery (e.g., complications relating to timing/onset of labor). |
| Induction / augmentation (oxytocin/ prostaglandin/ AROM) | O61 | Failed or abnormal induction of labor — important factor when analyzing trial of labor outcomes. |
| Analgesia/ anesthesia | O74 | Complications of anesthesia during labor — relevant for intrapartum management and maternal morbidity surveillance. |
| Mode of delivery (VBAC) | O80 | Outcome of delivery classification — central VBAC registry variable defining trial success or failure. |
| Mode of delivery (Instrumental Vaginal) | O81 | Outcome of delivery classification — central VBAC registry variable defining trial success or failure. |
| Mode of delivery (Emergency CS) | O82 | Outcome of delivery classification — central VBAC registry variable defining trial success or failure. |
| Mode of delivery (Elective CS) | O82 | Outcome of delivery classification — central VBAC registry variable defining trial success or failure. |
| Duration of labor (first/second stage) | O63 | Prolonged or obstructed labor — potential indicator for uterine rupture risk or need for emergency CS. |
| Intrapartum fever or chorioamnionitis | O75 | Infectious or inflammatory complications during labor — associated with neonatal sepsis and emergency CS. |
| Shoulder dystocia | O66 | Mechanical complications of labor — associated with trauma, emergency interventions, and postpartum hemorrhage. |
| Operative vaginal delivery | O81* | Delivery by forceps and vacuum extractor. This is an outcome of delivery classification, which is a central variable in registries like VBAC to define trial success or failure. |
| Estimated blood loss | O72 | Postpartum hemorrhage — major short-term outcome and key indicator in VBAC safety analysis. |

*O81.x depending on instrument.

## Section C: Short-term maternal outcomes (delivery to ≤6 Weeks)

| **Specific Condition** | **ICD-10 Code** | **Notes (Meaning and VBAC Relevance)** |
| --- | --- | --- |
| Postpartum hemorrhage (PPH ≥1000 mL or clinical diagnosis) | O72 | Specificity for time (immediate, delayed) is required. |
| Blood transfusion: Post-hemorrhagic anemia | D62 | (Acute post hemorrhagic anemia) is typically the reason for the transfusion. |
| Injury to Bladder | O71 | Obstetric trauma (other injury to pelvic organs). |
| Injury to Bowel | O71 | Obstetric trauma (other injury to pelvic organs). |
| Injury to Ureter | O71 | Obstetric trauma (other injury to pelvic organs). |
| Rupture/dehiscence (during labor) | O71 | Obstetric trauma. |
| Uterine atony | O62 | Abnormalities of forces of labor. |
| Status post-hysterectomy | Z90 | Acquired absence of uterus. |
| Endometritis | O86 | Infection of genital tract (puerperal). |
| Obstetric surgical wound infection | O86 | Infection of obstetric surgical wound. |
| Pulmonary embolism (PE) | O88 | Obstetric embolism. |
| Deep vein thrombosis (DVT) | O88 | Obstetric embolism. |
| Aftercare/Monitoring (LOS) | Z51 | Encounter for other specified aftercare. |
| General/Regional Anesthesia complication | O74 | Complications of anesthesia during labor/delivery. |
| Long-term (opioid) use | Z79 | Long-term current drug therapy. |
| Sepsis during Labor | O75 | Complications of labor and delivery |
| Puerperal Sepsis | O85 | Puerperal sepsis. |
| Obstetric shock | O75 | Shock during or following labor/delivery. |
| Acute renal failure | N17 | Acute kidney failure. The obstetric code O90 may also be used secondarily. |
| Pyrexia during labor | O75 | Pyrexia during labor. |
| Puerperal pyrexia (Unknown Origin) | O86 | Puerperal pyrexia of unknown origin. |
| Cystitis (Postpartum) | O86 | UTI following delivery. N30 may be primary. |
| Pyelonephritis (Postpartum) | O86 | UTI following delivery. N10 may be primary. |
| Urinary retention | R33 | Urinary retention. |
| Dehiscence of perineal wound | O90 | Complications of the puerperium, NEC. |
| Dehiscence of abdominal wound | O90 | Complications of the puerperium, NEC. |
| Breastfeeding complications | O92 | Other disorders of breast associated with childbirth. |
| Puerperal mental disorder (acute onset) | F53 | Severe mental/behavioral disorders associated with the puerperium. |
| Readmission ≤6 weeks; reason | O90 | Complications of the puerperium not elsewhere classified. This category is often used when a complication that arose postpartum (like wound dehiscence or persistent hypertension) requires a readmission or encounter. |
| Maternal death ≤42 days postpartum | O96 | Death from any obstetric cause, 43 days to 1 year after delivery (often used as underlying cause code). For ≤ 42 days, the underlying O-code (e.g., O72) is used. |

## Section D: Long-Term Maternal Outcomes (>6 Weeks to 12 Months)

| **Specific Condition** | **ICD-10 Code** | **Notes (Meaning and VBAC Relevance)** |
| --- | --- | --- |
| Urinary stress incontinence | N39 | Other disorders of urinary system. |
| Anal incontinence | R15 | Fecal incontinence. |
| Uterovaginal prolapse | N81 | Female genitals prolapse. |
| Chronic pelvic pain | N94 | Pain and other conditions associated with female genital organs. |
| CS-scar pain | R52 | Chronic pain, not elsewhere classified. |
| Pelvic adhesions (post-procedure) | N99 | Post procedural disorders of genitourinary system. |
| Paralytic ileus/ sub ileus | K56 | Paralytic ileus and intestinal obstruction without hernia. |
| Caesarean Scar Hernia | K43 | Ventral hernia. This category is used because a hernia occurring at the site of a prior surgical incision, such as a Cesarean Section scar, is classified as an incisional or ventral hernia. |
| Postpartum depression | F53 | Mental/behavioral disorders associated with the puerperium. |
| Anxiety disorder | F41 | Other anxiety disorders. |
| New hypertension (Persisting post-pregnancy) | O90 | (Maternal hypertension originating during pregnancy and persisting in the puerperium). |
| Persistent (Chronic/Pre-existing) hypertension | I10 | Essential (primary) hypertension. |
| Type 1 Diabetes Mellitus | E10 | Type 1 diabetes mellitus.  (post-pregnancy onset) |
| Type 2 Diabetes Mellitus | E11 | Type 2 diabetes mellitus.  (post-pregnancy onset) |
| Rheumatoid Arthritis (RA) | M05 | Seropositive rheumatoid arthritis. |
| Systemic Lupus Erythematosus (SLE) | L93 | Lupus erythematosus. |
| Multiple Sclerosis (MS) | G35 | Multiple sclerosis. |
| Placenta Previa | O44 | Placenta Previa. |
| In subsequent pregnancy | O43 | Disorders of placenta (morbidly adherent placenta). |
| Uterine rupture in subsequent pregnancy | O34 | Maternal care for known/suspected abnormality of pelvic organs. |
| Subsequent conception | O00 | Ectopic pregnancy. |
| Subsequent conception | O03 | Spontaneous abortion. |
| Stillbirth (subsequent pregnancy) | P95 | Fetal death of unspecified cause(this is the fetal outcome code). |
| Infertility after index pregnancy | N97 | Female infertility. |
| Peripartum hysterectomy (subsequent pregnancy) | Z90 | Acquired absence of organ. |
| Abnormal placentation care pathway | O43 | Maternal care for known or suspected fetal problems. |
| Pelvic adhesions (post-procedure) | N99 | Post procedural disorders of genitourinary system. |
| Maternal death ≥1 year postpartum | O97 | Sequelae of direct obstetric causes. |

# References:

1. World Health Organization. International Statistical Classification of Diseases and Related Health Problems, 10th Revision (ICD-10). Geneva: World Health Organization; 2019. Available from: <https://icd.who.int/browse10/2019/en>
2. Sandall J, Tribe RM, Avery L, et al. Short-term and long-term impacts of caesarean section on women’s and children’s health. The Lancet. 2018;392(10155):1349–1357.
