# Supplementary material for: Clinical, psychosocial and cognitive outcomes following VBAC: a protocol for a sub-national cohort study in Saudi Arabia (2019–2024)
Source: BMJ Open. 2026 Oct 13;16(10):e114754. doi: 10.1136/bmjopen-2025-114754 (PMC13663696; doi:10.1136/bmjopen-2025-114754)
Supplement: Supplementary file 4 [file bmjopen-16-10-s003.docx]

**Supplementary file 3. Child outcomes and ICD-10 codes**

## Section A -Short-term child outcomes (0–3 years)

| **Specific condition** | **ICD-10 Code** | **Notes** |
| --- | --- | --- |
| Perinatal death (excluding lethal congenital anomalies) | P95 | Broad code for fetal/perinatal death of unspecified cause; key short-term safety indicator in delivery mode comparisons. |
| Stillbirth | P95 | Fetal death of unspecified cause; relevant for timing of delivery and intrapartum management. |
| Neonatal death (<7 days) | P96 | Broad neonatal mortality category when specific cause is varied or unspecified. |
| Neonatal Respiratory Distress Syndrome (RDS) | P22 | Surfactant deficiency; more common in elective CS without labor exposure. |
| Persistent Pulmonary Hypertension of the Newborn (PPHN) | P29 | Failure of circulatory transition after birth; linked with elective CS. |
| Pneumothorax | P25 | Air leak syndrome; occurs with severe respiratory distress or ventilation. |
| Massive pulmonary hemorrhage | P26 | Severe respiratory compromise; associated with prematurity and infection. |
| Meconium aspiration syndrome | P24 | Aspiration of meconium-stained fluid; associated with fetal distress. |
| Atopy (early allergy) | L20 | Atopy does not have a standalone ICD-10 code; L20 (atopic dermatitis) is used to represent atopy-related immune/allergic phenotypes. Early allergic manifestations; CS-linked immune alterations. |
| Asphyxia (general) | P21 | Impaired oxygenation at birth; major contributor to early morbidity. |
| Medium asphyxia (Apgar 5 min <4) | P21 | Severe perinatal hypoxia; key predictor of neurological injury. |
| Neonatal seizures ('cramps') | P90 | The term ‘cramps’ refers to seizure-like neonatal movements classified under P90 (Neonatal seizures). Seizures due to asphyxia, metabolic issues, or trauma. |
| Hypoxic-ischemic encephalopathy (HIE) Grade III | P91 | Severe neonatal encephalopathy; high risk of long-term disability. |
| Intracranial hemorrhage | P52 | Hemorrhage due to trauma, asphyxia, or prematurity. |
| Basal skull fracture (birth trauma) | S02 | Birth trauma–related fracture; associated with difficult delivery. |
| Subgaleal hematoma | P12 | Severe extracranial bleeding linked to vacuum extraction. |
| Hypoglycemia requiring treatment | P70 | Metabolic instability common post-CS or in IDMs. |
| Infections  (neonatal meningitis) | G03 | CNS infection with high risk of long-term impairment. |
| Infections  (Neonatal pneumonia) | P23 | Acute lung infection linked with aspiration or prematurity. |
| Infections  (Neonatal sepsis) | P36 | Early-onset bacterial infection. |
| NICU admission >4 days | Z76 | Indicator of significant neonatal morbidity. |
| Altered immune development | D84 | Broad immunological disorder; CS-linked immune differences. |
| Reduced intestinal microbiome diversity | B99 | Broad dysbiosis category; CS birth affects microbial colonization. |
| Altered microbiome | B99 | Reflects disruption of normal microbial exposure with CS delivery. |
| Blunted neonatal stress response | R68 | Reduced cortisol surge; metabolic & immune implications. |
| Allergies | L20 | Allergic/atopic immune tendencies linked to altered neonatal microbiome after cesarean delivery. |
| Iatrogenic prematurity | P07 | Prematurity due to mis-timed elective CS. |

## Section B — Long-term child outcomes (>3 Years)

| **Specific condition** | **ICD-10 Code** | **Notes** |
| --- | --- | --- |
| Long-term asphyxia | P91 | Severe neonatal encephalopathy predicts long-term impairment. |
| Cerebral palsy | G80 | Permanent motor disorder due to perinatal brain injury. |
| Epilepsy | G40 | Chronic seizure disorder linked to neonatal injury. |
| Autism spectrum disorder  (Including Asperger) | F84 | Neurodevelopmental condition; higher incidence reported after CS. |
| ADHD | F90 | Behavioral/neurodevelopmental condition. |
| Childhood mental disorders | F99 | Broad category for emotional/behavioral conditions. |
| Multi-handicap | F73 | Severe intellectual disability with multiple impairments. |
| Asthma | J45 | Higher rates observed in CS-born children. |
| Allergy / chronic atopy | L20 | Persistent allergic tendency. |
| Type 1 diabetes mellitus | E10 | Autoimmune diabetes linked to alter early immune priming. |
| Celiac disease | K90 | Immune-mediated gluten intolerance. |
| Inflammatory bowel disease | K52 | Chronic gut inflammation. |
| Atopy (chronic) | L20 | Chronic allergic phenotype. |
| Failure to thrive | R62 | Growth faltering beyond infancy. |
| Type 2 diabetes (childhood) | E11 | Metabolic condition influenced by early growth patterns. |
| Childhood obesity | E66 | Higher BMI trajectories reported in CS-born children. |
| Chronic malnutrition | E44 | Long-term nutritional insufficiency. |
| Gastrointestinal disorders | K92 | Chronic or functional GI symptoms. |
| Childhood cancers | C80 | Broad unspecified malignancy category. |
| Death (after neonatal period) | R99 | Unspecified childhood mortality. |
| Emotional/behavioral/cognitive/  educational  difficulties-outcomes | F81 | Learning and developmental challenges. |

# References

1.World Health Organization. International Statistical Classification of Diseases and Related Health Problems, 10^th^ Revision (ICD-10). Geneva: World Health Organization; 2019. Available from: <https://icd.who.int/browse10/2019/en>.

2.Sandall J, Tribe RM, Avery L, Mola G, Visser GH, Homer CS, et al. Short-term and long-term impacts of caesarean section on children’s health. The Lancet. 2018;392(10155):1349–1357.
